# Improving cardiac myocytes performance by carbon nanotubes platforms^†^

**DOI:** 10.3389/fphys.2013.00239

**Published:** 2013-09-03

**Authors:** Valentina Martinelli, Giada Cellot, Alessandra Fabbro, Susanna Bosi, Luisa Mestroni, Laura Ballerini

**Affiliations:** ^1^Molecular Medicine Group, International Centre for Genetic Engineering and BiotechnologyTrieste, Italy; ^2^Department of Neurobiology, International School for Advanced StudiesTrieste, Italy; ^3^Department of Life Sciences, University of TriesteTrieste, Italy; ^4^Department of Chemical and Pharmaceutical Sciences, University of TriesteTrieste, Italy; ^5^Cardiovascular Institute, University of ColoradoDenver, CO, USA

**Keywords:** nanotechnology, synthetic interface, cardiomyocyte maturation, cardiomyocyte proliferation, cardiac tissue engineering

## Abstract

The application of nanotechnology to the cardiovascular system has increasingly caught scientists' attention as a potentially powerful tool for the development of new generation devices able to interface, repair, or boost the performance of cardiac tissue. Carbon nanotubes (CNTs) are considered as promising materials for nanomedicine applications in general and have been recently tested toward excitable cell growth. CNTs are cylindrically shaped structures made up of rolled-up graphene sheets, with unique electrical, thermal, and mechanical properties, able to effectively conducting electrical current in electrochemical interfaces. CNTs-based scaffolds have been recently found to support the *in vitro* growth of cardiac cells: in particular, their ability to improve cardiomyocytes proliferation, maturation, and electrical behavior are making CNTs extremely attractive for the development and exploitation of interfaces able to impact on cardiac cells physiology and function.

## Introduction

The development of innovative materials able to effectively interface biological tissues and to tune and boost their functional performance is an active and heterogeneous field of current research, and nanotechnological approaches for interfacing excitable cells are disclosing very promising perspectives (Silva, 2006; Place et al., 2009; Dvir et al., 2011a,b; Chan et al., 2013; Shin et al., 2013). In particular, the development of scaffolds for cardiac tissue engineering based on conductive nanomaterials is raising growing interest (Chan et al., 2013; Shin et al., 2013); in this scenario, the exploitation of carbon nanotubes (CNTs) technology to the development of devices able to interface and support cardiac physiology is emerging as a concrete, exciting perspective. Indeed, the electrical conductivity of the material is an important factor to design effective scaffolds able to interface to excitable cells (Dvir et al., 2011b; Chan et al., 2013).

CNTs are emerging as an ideal material to be interfaced with electrically active tissues as neuronal and cardiac tissues. This relatively recently discovered class of materials possess high electrical and thermal conductivity, strong mechanical resistance and elasticity and have demonstrated good biocompatibility in many biological studies. Moreover it has been shown that the functionalization of CNT walls can tune their biological properties and their metabolic fate (Singh et al., 2006; Lacerda et al., 2008a,b). Based on these motivations several hypothetical biomedical applications involving CNT technology have been put forward. Among them, one of the more appealing is in the area of tissue engineering. Thus, in recent years, CNTs have been largely studied as key components for innovative composite materials in tissue engineering.

Highly conductive CNTs-based scaffolds are able to support the growth and to profoundly impact on and boost the functional performance of neuronal excitable cells (Sucapane et al., 2009; Fabbro et al., 2011; Voge and Stegemann, 2011). On the contrary, little is known about the impact of CNT-based platforms on muscular cells in general and on cardiomyocytes in particular. However, recently, the idea of applying this innovative nanomaterial to impact on and tune cardiac performance emerged: interfacing CNTs-based scaffolds to highly specialized and terminally differentiated cardiac myocytes might indeed represent a promising strategy for the development of two- and three-dimensional nanostructured, transplantable cell-enrichable tissue implants (Dvir et al., 2011a,b; Chan et al., 2013).

This Mini Review will summarize the current state-of-the-art of the application of CNTs technology-based scaffolds to cardiac cells physiology and function *in vitro*, highlighting any promising perspective toward functional improvement of cardiac tissue.

## Effect of CNTs on myocytes electrical behavior

The impact of pure CNTs platforms on the electrophysiological behavior of developing cardiomyocytes has been characterized for the first time by Martinelli et al. (2012). In this study, defunctionalized highly purified carbon-nanotube scaffolds were used as substrates to culture neonatal rat ventricular myocytes (NRVMs); after 2–3 days in culture, the electrical properties of single myocytes, still not fused to form multinucleated syncytia, were analyzed by the patch clamp technique. NRVMs grown on CNTs layers showed more negative values of resting membrane potential than controls (i.e., NRVMs grown on gelatin substrates), while other passive membrane properties, such as cell capacitance and input resistance, were comparable in cells developed on CNTs or gelatin. Similarly, multinucleated cells able to contract and originating from the fusion of many single NRVMs exhibited a more negative resting potential when grown on CNTs layer compared to controls, with no effect on other basic properties (capacitance and input resistance; Martinelli et al., 2013). The same study also investigated *active* electrical properties of cardiomyocytes and revealed that CNTs substrates increase the fraction of NRVMs able to fire action potentials, while preserving action potentials kinetic and firing frequency (Martinelli et al., 2012). The impact of CNTs on cardiomyocytes electrical activity patterns was further investigated by calcium imaging experiments, to monitor the appearance of action potential-independent spontaneous calcium transients in NRVMs. In both controls and CNTs-interfaced cultures, intracellular calcium oscillatory behavior was pretty heterogeneous. However, NRVMs showing either only sporadic and isolated events, or clusters of slow frequency events, or clusters of fast events could be distinguished. When cultures were grown on CNTs, a larger fraction of cells exhibiting sporadic events compared to controls emerged, confirming that interfacing to CNTs promotes a more rapid NRVMs maturation (Martinelli et al., 2013).

**Figure 1:**
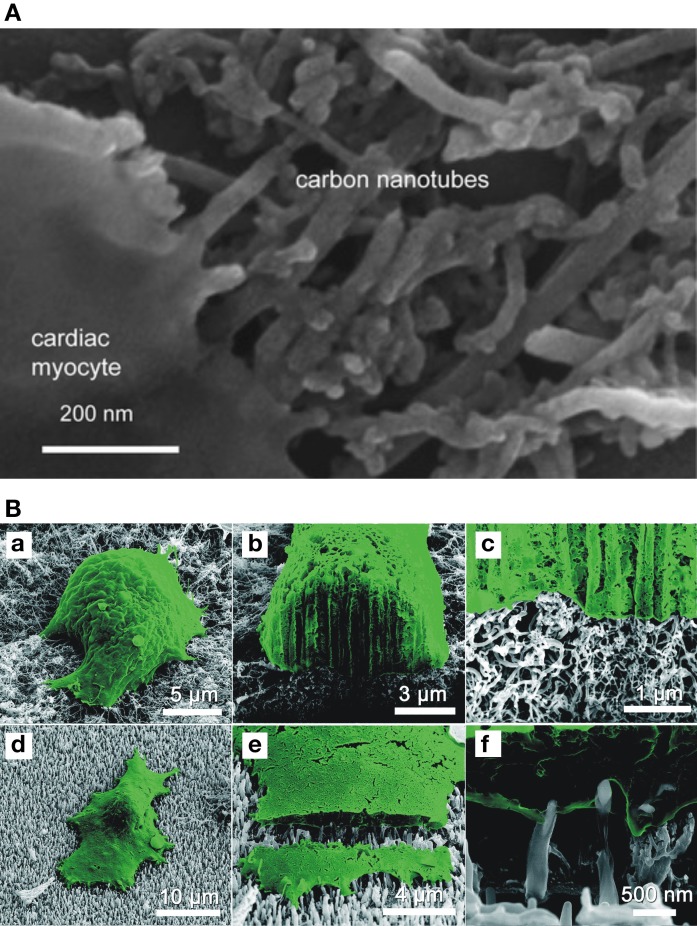
**Cardiomyocytes intimately hybridize with CNTs substrates. (A)** Scanning electron micrograph showing the close interactions between the membrane of a cultured cardiomyocyte and the CNTs growth substrate. Reprinted with permission from Martinelli et al. (2013). **(B)** Scanning electron micrographs of myocytes (in false color) cultured on randomly-oriented CNTs (a–c) or on vertically-aligned CNTs (d–f). On the vertically aligned substrate, single CNTs cross the membrane and penetrate into the intracellular space. Reprinted with permission from Fung et al. (2010).

The more hyperpolarized resting potential detected in both single NRVM and multinucleated syncytia, and the higher percentage of cells able to fire action potential and showing sporadic calcium oscillations indicate that the process of maturation of cardiac cells is accelerated by CNTs, while such properties are kept in a physiological range. However, the mechanisms underlying this accelerated development have not been clarified yet, although the tight physical interactions between cellular membrane and CNTs, revealed by electron microscope analyses, are likely to play a key role (Martinelli et al., 2012, 2013; Figure 1). In this respect, it is interesting to note that these cardiomyocytes/CNTs hybrids are very similar to what has been observed for other excitable cells (neurons) grown on CNTs supports (Cellot et al., 2009), thus, indicating an intrinsic CNTs ability to intimately interact with excitable cells membranes.

The unique properties of CNTs as interfaces for cardiac tissue growth has been recently implemented by integrating conventional scaffolds materials with CNTs, to create high-performance hybrid scaffolds. Shin et al. (2013) reported the use of a new scaffold (CNT-GelMA) composed of CNTs homogenously incorporated into gelatin methacrylate (GelMA): the two components together form a biocompatible, mechanically strong, and electrically conductive structure. Neonatal cardiomyocytes were cultured for several days on such scaffold; interestingly, cells grown on CNT-GelMA are characterized by a more stable spontaneous beating behavior, with a beating rate significantly higher than that of cells grown on GelMA alone. Moreover, the cardiac tissue appears more mechanically strong and intact on CNT-GelMA than on GelMA alone, probably because of a better tissue anchoring to the CNT-reinforced scaffold structure, as indicated by the physical contacts between cell membranes and carbon nanotube-nanofibers observed by electron microscopy. Importantly, CNT-GelMA scaffold seems to exert also a protective effect for host cells. Indeed, treatment with heptanol, able to quickly alter the physiological beating in control cells acting on gap junctions, has only a delayed, attenuated effect on cardiac tissue cultured on CNT-GelMA. This finding suggests that CNTs might promote the electrical communication between cardiac cells possibly beyond gap junctions' efficacy (Shin et al., 2013).

## Impact of CNTs scaffolds on myocytes morphology, proliferation, and maturation

The boost toward a functionally and electrically more mature phenotype induced by CNTs-based scaffolds is likely to be related to a general impact of CNTs substrates on cardiomyocyte growth, including a modulation of their morphology and proliferative ability. Recently, Martinelli et al. (2012) demonstrated the ability of pure multi-walled carbon nanotube scaffolds to promote cellular viability, myocyte proliferation, and maturation in NRVMs, compared to control. In this study, metabolic activity assays, bromodeoxyuridine (BrdU) incorporation, flow cytometry and cell division markers demonstrated that CNTs not only support cardiomyocytes viability, but also selectively influence NRVM proliferating ability, with a doubling in cell number after 3 days culturing. Importantly, CNTs impact on cell proliferation was selective for cardiomyocytes, as fibroblast viability and proliferation were unchanged. Furthermore, CNTs growth substrates ability to promote cardiomyocytes proliferation is significantly higher compared to other growth-permissive substrates [indium tin oxide—ITO-glass, gelatin-coated ITO-glass, amorphous carbon coated glass, and poly (D,L-lactide-co-glycolide)—PLGA-] (Martinelli et al., 2012).

Similarly, Meng et al. (2013) showed that human cardiomyocytes improve their adhesion and proliferation when cultured on a composite of carbon nanofibers and poly-2-hydroxyethyl methacrylate (pHEMA, a biocompatible hydrogel) compared to pHEMA alone or tissue culture polystyrene, an effect strictly dependent on carbon nanofibers concentration (Meng et al., 2013).

**Figure 2:**
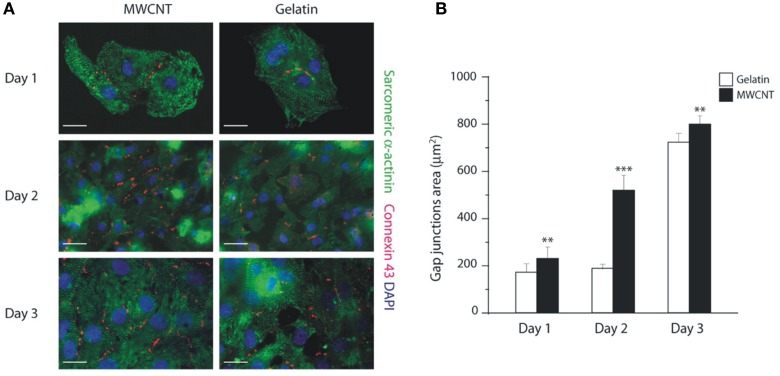
**Increased expression of gap junctions in cardiomyocytes grown on CNTs scaffolds. (A)** Neonatal cardiomyocytes grown on CNTs (MWCNT, left) or on control gelatin substrate (right) stained for connexin 43 (red), sarcomeric Alpha-Actinin (green), and counterstained with DAPI (blue). Bars: 50 μm. **(B)** The expression of connexin 43 (gap-junctions) after 1, 2, and 3 days culturing is increased in cultures grown on the CNTs scaffold. Reprinted with permission from Martinelli et al. (2013). ^**^p < 0.01; ^***^p < 0.001.

In addition to cardiomyocytes proliferative ability, CNTs platforms also have a profound effect on cardiomyocytes gene program and syncytia development. This ability has been recently characterized by Martinelli et al. (2013), by studying the expression of five key genes involved in the growth/fetal gene expression program: cardiac beta-myosin heavy chain (β MHC), alpha-myosin heavy chain (α MHC), A-type Natriuretic Peptide (ANP), sarcoplasmic reticulum Ca^2+^ ATPase2a (SERCA2a) and skeletal-Actin (Sk-Actin). Genes associated to a more mature phenotype like α MHC are significantly increased in cardiomyocytes grown on CNTs compared to gelatin controls after 3 days culturing, while at the same time, the levels of pathological cardiac hypertrophy markers (β MHC and Sk-Actin) were unaffected. Therefore, after an initial pro-proliferative profile, CNTs scaffolds promote a “mature gene expression profile” of cardiomyocytes over time (Martinelli et al., 2012, 2013). Importantly, the amount of gap-junctions (revealed by Connexin43 immunostaining; Figure 2) was significantly higher on CNTs than on control substrate, in agreement with the significant increase in gap junctions in syncytia-beating domains (Martinelli et al., 2012, 2013).

Pure CNTs growth supports also show another very interesting and unique property, i.e., their protective activity against cardiomyocytes hypertrophy, induced by the G-protein-coupled receptor ligand phenylephrine (Martinelli et al., 2013). These findings further emphasize the potential of CNTs for a variety of therapeutic applications in heart diseases, including tissue engineering and stem cells-based therapies.

In tissue engineering, the search for new biomechanical supports for cell growth to promote the development of functional tissue is a subject of extraordinarily intense research activity, and many groups have introduced novel strategies to study cell adhesion, viability, proliferation and organization on highly porous two- and three-dimensional scaffolds (Langer and Vacanti, 1993; Dvir et al., 2011a,b; Chan et al., 2013; Shin et al., 2013). In this context, CNTs have attracted tremendous attention: indeed, significant advancements have been recently achieved by the excellent mechanical integrity and advanced electrophysiological functions of the CNTs-based cardiac constructs developed by Shin et al. (2013; see above). Their CNT-GelMA hydrogel indeed strongly improves adhesion, viability, proliferation, and organization of the engineered cardiac tissue compared to control GelMA hydrogel films. On CNT-GelMA cardiomyocytes are homogeneously distributed and better interconnected, covering the entire CNT-GelMA surface with a strong increase in cell adhesion and elongation, spreading, retention and viability compared to GelMA controls. The 2D cardiac tissue cultured on CNT-GelMA also displays better defined and elongated sarcomeric structures with partial uniaxial alignment, with an up-regulation of the cardiac specific markers Sarcomeric α-Actinin and Troponin I (with only a moderate Cx-43 expression). Importantly, similarly to pure CNTs scaffolds (Martinelli et al., 2012), CNT-GelMA substrates do not alter cardiac fibroblasts proliferation (Shin et al., 2013).

It is important to note that, in addition to cardiac myocytes, the biocompatibility and impact of CNTs scaffolds on cellular growth and differentiation have been also demonstrated for non-cardiac muscle cells. CNT-GelMA, in fact, is also able to support the growth of C2C12 skeletal myoblasts (Tsukahara and Haniu, 2011). Ramón-Azcón et al. (2013) exploited dielectrophoresis (electric field application to induce molecules charge polarization) to pattern CNTs into GelMA hydrogels. Thanks to this approach, the authors were able to generate CNT-GelMA scaffolds in which the nanomaterial is highly aligned, a property conferring better conductivity to the scaffold. Skeletal myoblasts grown on these substrates showed a marked alignment and boosted expression of myogenin, MRF4, sarcomeric actin, α-Actinin and myosin heavy chain isofactor IId/x compared to cells grown on GelMa hydrogel with dispersed CNTs, i.e., a more mature profile of contracting cells. Similarly, Holt and coworkers (2013) observed that highly aligned CNTs encourage the growth of mouse precursor skeletal muscle cells along a preferred direction.

McKeon-Fischer et al. (2011) showed that, when cultured on an acid-functionalized multi-walled carbon nanotube/polycaprolactone electrospun scaffold (PLC-MWCNT-H), primary skeletal muscle cells display an increased ability to form large multinucleated constructs with increased actin filaments interaction compared to polycaprolactone scaffolds alone. Furthermore, MacDonald et al. (2005) showed that collagen-CNTs constructs support smooth muscle fibers growth. CNTs are therefore a versatile and effective material to implement and strengthen the biological activity of a variety of composite tissue scaffolds for the growth and functional differentiation of various muscular cell types.

In addition to CNTs-based growth-supporting scaffolds, the impact of the application of CNTs in the form of suspension in the culture medium on muscular (including cardiac) cells survival and growth has been investigated. In 2006, Garibaldi and colleagues exposed cardiac muscle cells from the H9c2 (2-1) heart cell line to pure single-walled nanotubes suspended in culture medium, and observed no short-term toxicity of soluble CNTs (limited proliferative ability and morphological changes were identified only after CNTs-treated cells reseeding; Garibaldi et al., 2006). Recently, Lin et al. (2012) reported that dispersed suspension of pure single-walled CNTs in the culture medium promotes vascular adventitial fibroblasts transformation into myofibroblasts, with an up-regulation of the SM_22−α_ differentiation marker expression, although accompanied by an increased oxydative damage (Lin et al., 2012). It has to be noted, however, that pure (non-functionalized) CNTs are a water-insoluble, aggregate-forming material, and the presence of CNTs aggregates is related to decreased cell viability, at least for smooth muscle cells (Raja et al., 2007).

## CNTs for cardiac cells electrical interfacing

In addition to their use as cell-instructive and growth-supporting conductive scaffolds, the unique CNTs electrical properties suggested their exploitation for the development of innovative nanoengineered devices to both stimulate and record electrical activity from electrically active cells such as muscular ones, improving signal-to-noise ratio, and causing minimal cell damage, similarly to what has been shown for neuronal CNTs-based interfaces (Fabbro et al., 2011; Bareket-Keren and Hanein, 2012). In 2010, Fung and colleagues interfaced NRVMs to microfabricated arrays of CNT microelectrodes: in addition to an extremely low electrode impedance of both pristine and O_2_ plasma-treated CNTs microelectrodes, the authors showed that the interaction of NRVMs membrane with the electrodes strictly depends on CNTs orientation: while vertically-aligned nanotubes penetrate NRVMs membrane, randomly oriented ones remain external to the cells, suggesting CNTs microelectrodes as effective tools to strongly improve nano/bio interfaces (Figure 1). Accordingly, Nick et al. (2012) reported the use of an array of multiple multielectrodes covered by CNTs, where chicken embryonic cardiomyocytes were cultured and their electrical activity monitored. Cardiomyocyes grew well on CNTs-integrated arrays, forming a dense cellular network and starting to contract after about 36 h from plating. Recordings made by CNTs-covered electrodes were characterized by a lower noise level and a better signal resolution compared to recordings obtained by standard gold or titanium electrodes (Nick et al., 2012). Similarly, multielectrode arrays can be covered by a CNTs and poly (3,4-ethylenedioxythiophene) (PEDOT, a conductive polymer) composite (Gerwig et al., 2012). In this work, primary embryonic cardiomyocytes were cultured for up to 10 days on PEDOT alone or on PEDOT-CNTs microelectrodes. Cells in both culturing conditions displayed excellent viability and functionality, as they started to contract 2–3 days after plating and formed a two dimensional syncytium on both PEDOT and PEDOT-CNTs microelectrode arrays. Also in this case CNTs enhanced the electrodes performance, as PEDOT-CNTs microarrays have higher capacitance and charge injection capacity than electrodes covered by PEDOT alone, while both type of microarrays are effective in showing higher signal-to-noise ratio compared to titanium electrodes (Gerwig et al., 2012).

A very interesting application of the above reported CNTs-based growth scaffolds is the possibility of eliciting contractions of the cultured cardiac tissue by applying an electric field to the growth scaffold itself. Indeed, thanks to their excellent conductive properties, when CNTs are integrated in the growth scaffold structure the intensity of the electrical field necessary to elicit contraction is hugely reduced compared to non-CNTs-containing scaffolds (as in the case of CNT-GelMA composites: Shin et al., 2013). This property is extremely important in the perspective of employing CNTs-based devices for cardiac tissue stimulation purposes *in vivo*, as it preserves cardiac cells from the damage induced by the pH and gas environment alterations possibly produced in the ionic solution by the electrical stimulation itself (Shin et al., 2013).

Electrical stimulations delivered through the CNTs-containing scaffolds can also be exploited to control and tune the maturation of both cardiac and skeletal muscular cells. Mooney et al. (2012) observed that the electrical stimulation of human mesenchimal stem cells (MSCs)cultured on a poly-L-lactide acid (PLA)-CNT scaffold show a more elongated morphology compared to unstimulated MSCs. Electrical stimulation also impacts on CNTs-exposed MSC differentiation toward a cardiomyocyte lineage, as it modulated the expression of the cardiac marker CMHC (cardiac myosin heavy chain; Mooney et al., 2012). Interestingly, the electrical stimulation of MSCs exposed to *soluble* COOH-functionalized CNTs pushes their differentiation toward a cardiac lineage, as the treatment induced a strong up-regulation of the cardiac markers NKx2.5, GATA-4 and CTT (Mooney et al., 2012).

The combination of CNTs-based scaffolds and electrical stimulation has been applied also to skeletal muscle cells. Sirivisoot and Harrison (2011) reported that skeletal myotubes cultures grown on a polyurethane-CNTs composite scaffold and electrically stimulated through the scaffold itself, show a higher number and length of cells compared to cultures grown without electrical stimulation. Furthermore, in the presence of electrical stimulation, myotube formation increased in the polyurethane-CNTs composite compared to polyurethane alone.

## Remarks and conclusions

CNTs intimately interact with cardiomyocytes membranes, a property allowing a strong improvement of the electrophysiological recordings obtained through CNTs-based devices. On the other hand, scaffolds based on this nanomaterial (pure or mixed with biocompatible polymers) can boost, within a physiological range, cardiac cells maturation, while supporting and promoting cellular viability and adhesion, stimulating a pro-proliferative program and promoting cell differentiation. Furthermore, CNTs scaffolds are able to exert a protective role for cardiomyocytes against pathological stimuli (like pathological hypertrophy). All these properties make CNTs excellent candidates for the development of innovative, effective, and performing devices to interface and engineer cardiac tissue, with the added value of the intrinsic electric conductivity of the scaffold itself. The promising exploitation of CNTs-based platforms for cardiac applications is further encouraged by the growing amount of data on CNTs biocompatibility and impact on immune responses. Serious concerns have been raised about CNTs safety as toxic effects were reported after CNTs injection in lungs (Poland et al., 2008). CNTs toxicity is, however, related to the presence of impurities (Vittorio et al., 2008) and to CNTs geometry and functionalization (Sayes et al., 2006; Bianco et al., 2011; Al-Jamal et al., 2012). Appropriately functionalized CNTs do not impact on B and T lymphocytes and macrophages viability (Dumortier et al., 2006). Subcutaneously applied oxidized MWCNTs induce complement activation and proinflammatory cytokines production which disappear over time, with only minor inflammatory reactions at the injection sites (Meng et al., 2011). Furthermore, implanted devices based on immobilized CNTs induce only minimal local foreign-body responses (Nayagam et al., 2011). Appropriate CNTs manipulation can thus powerfully decrease or eliminate their potential adverse effects and increase their safety of use.

### Conflict of interest statement

The authors declare that the research was conducted in the absence of any commercial or financial relationships that could be construed as a potential conflict of interest.
